# Cardiac Edema Is Associated with White Matter Hyperintensities in Patients with Inflammatory Arthritides: A Combined Brain/Heart MRI Study

**DOI:** 10.3390/jcm14113726

**Published:** 2025-05-26

**Authors:** George Markousis-Mavrogenis, Aliki Venetsanopoulou, Ioannis Ntalas, Ioannis Pagounis, Christina Naka, Dionisis Toliopoulos, Dimitrios Apostolou, Paraskevi Voulgari, Sophie I. Mavrogeni

**Affiliations:** 1 University Research Institute of Maternal and Child Health and Precision Medicine and UNESCO Chair in Adolescent Health Care, Medical School, National and Kapodistrian University of Athens, Aghia Sophia Children’s Hospital, 11527 Athens, Greece; georgemm32@gmail.com; 2 Rheumatology Department, University of Ioannina, 45110 Ioannina, Greece; a.venetsanopoulou@gmail.com (A.V.); pvoulgari@uoi.gr (P.V.); 3 IANO Epirus Diagnostic Laboratory, 45500 Ioannina, Greece; dr.ntalas@gmail.com (I.N.); pagounisgi@gmail.com (I.P.); chrisnaka13@gmail.com (C.N.); diotoliopoulos@yahoo.com (D.T.); 4 Mediterraneo Hospital, 16675 Athens, Greece; apostolou@mediterraneohospital.gr; 5 Onassis Cardiac Surgery Center, 17674 Athens, Greece

**Keywords:** brain magnetic resonance imaging, heart magnetic resonance imaging, autoimmune inflammatory joint diseases, myocardial inflammation, myocardial fibrosis

## Abstract

**Background**: Inflammatory arthritides (IAs) are systemic inflammatory syndromes that can affect diverse body tissues. Central nervous system involvement has been reported, but is considered rare. We investigated the relationship between cardiac and subclinical brain involvement in patients with IAs. **Methods**: We consecutively enrolled 25 patients with IAs and 31 as disease controls with non-autoimmune cardiovascular diseases (CVDs) reporting cardiac symptoms. Each participant underwent combined brain/heart magnetic resonance imaging (MRI). We also recruited 25 consecutive asymptomatic healthy controls without CVDs who underwent brain MRI. MRI scans were performed on a 1.5 T system. We investigated cardiac function/tissue characterization and the presence/localization of white matter hyperintensities (WMHs). **Results**: All groups had similar ages (*p* = 0.267), and 16 (64%) patients with IAs vs. 7 (23%) disease controls vs. 16 (64%) healthy controls were women (*p* = 0.001). WMHs were detected in ≥1 brain area in 15 (60%) patients with IAs and 16 (53%) disease controls (*p* = 0.620). WMHs were significantly less prevalent amongst healthy controls [two (8%)] compared to patients with IAs (*p* < 0.001). Amongst patients with IAs, an increased cardiac T2 ratio was associated with an increased probability of WMH occurrence [OR per 0.1 unit change (95% CI): 1.29 (1.05–1.59), *p* = 0.016], while a higher cardiac T2 ratio (per 0.1 unit change) and extracellular volume fraction (ECV) were associated with higher WMH lesion burdens [β (95% CI): 0.12 (0.03–0.20), *p* = 0.008 and 0.25 (0.00–0.49), *p* = 0.049, respectively]. **Conclusions**: Patients with IAs and cardiac symptoms had significantly higher subclinical WMH burdens compared to age/sex-matched healthy controls. Myocardial edema was associated with a greater WMH burden, potentially suggesting shared pathophysiologic substrates.

## 1. Introduction

Inflammatory arthritides (IAs) are a group of systemic inflammatory syndromes that can affect diverse body tissues beyond the joints, including the blood vessels and the heart [1]. Overt central nervous system involvement has been reported as well, although it is considered rare [2,3,4]. Epidemiologic evidence has also demonstrated that patients with IAs such as rheumatoid arthritis (RA) are at increased risk of stroke [5,6] and die prematurely due to cardiovascular disease (CVD) [7,8]. Furthermore, they may present early evidence of cognitive dysfunction [9,10]. A systematic review and meta-analysis of cerebrovascular disease in autoimmune rheumatic diseases (ARDs) showed that the risk of any stroke is higher in patients with most types of autoimmune rheumatic diseases than it is in the general population [11]. In particular, patients < 50 years old with RA and systemic lupus erythematosus (SLE) were at an increased risk of ischemic and hemorrhagic stroke, by 60% to 100%, relative to the general population [11]. Thus, beyond overt central nervous system manifestations, there appears to be an increased prevalence of both CVD and cerebrovascular disease in patients with IAs.

The underlining cause behind CVD/cerebrovascular disease in patients with IAs is systemic inflammation [11,12]. The brain and the heart share significant similarity in their vascular anatomy and function, with both rely on deep penetrating arteries arising from conduit arteries on their surfaces for tissue perfusion [13,14,15,16]. Therefore, small vessel disease (SVD) can involve the penetrating vessels of both organs, leading to the dysfunction of their respective perfusion domains [1,2,3]. Nevertheless, even though similar mechanisms may drive cardiac inflammation and neuroinflammation [11], there are currently few data regarding the interaction between heart and brain involvement in patients with IAs other than their preponderance for co-occurrence.

The biological understanding of brain–heart interactions is further complicated by the inherent difficulty of obtaining tissue samples for analysis, particularly from the brain. In this regard, combined brain–heart (MRI) can offer significant advantages, as it has various established and well-validated uses in the evaluation of brain structure and function, while allowing the simultaneous evaluation of cardiac function and tissue properties [11]. In addition, MRI does not rely on ionizing radiation and can evaluate the brain and the heart in the same examination.

We hypothesized that patients with IAs and cardiac symptoms would show more pronounced evidence of brain lesions compared to healthy individuals, and that cardiac inflammation would be associated with brain lesions. To further evaluate this hypothesis, we examined a cohort of patients with IAs as well as two independent control cohorts (disease controls with cardiovascular symptoms and asymptomatic healthy controls) using combined brain–heart MRI.

## 2. Materials and Methods

### 2.1. Patients and Control Groups

We prospectively included 25 patients with IAs as well as 31 age-matched disease controls with non-autoimmune CVD, as described in our previous publication [11]. All patients were referred for cardiac MRI due to the presence of cardiac symptoms including shortness of breath, chest pain, and/or palpitations. In addition, no patients reported overt neurologic symptoms at the time of study inclusion. Upon obtaining informed consent for inclusion to this study as per the declaration of Helsinki, all patients underwent a comprehensive clinical neurologic evaluation to exclude dementia, memory loss, and/or cognitive dysfunction, and a complete brain MRI was carried out immediately following the cardiac MRI evaluation.

To evaluate the prevalence of white matter hyperintensities in a healthy general population, we also recruited an independent cohort of 25 age- and sex-matched individuals without cardiac or neurologic symptoms, who underwent a brain MRI study on a volunteer basis.

Patients that were aged <18 years, those with contraindications to MRI, including non-MRI compatible devices and/or metallic clips, and those with renal impairment were excluded from this study. All participants were consecutive, were enrolled over a period of 2 years, and provided written informed consent before study inclusion. The study protocol was approved by the local medical ethics committee before implementation.

### 2.2. Combined Brain/Heart Magnetic Resonance Imaging

Combined brain–heart MRI was performed in patients with IAs and disease controls using a 1.5 T system. In the case of healthy controls, an isolated brain MRI study was conducted.

The combined brain/heart MRI methodology protocols used in this study have been previously described in more detail [11]. We should emphasize that in this paper, different disease percentages were shown by the population of the current study compared to the study population of the 2020 publication [11].

Briefly, the cardiac MRI protocol included standard steady-state free-precession cine MRI for the evaluation of biventricular volumes and ejection fractions. The evaluation of cardiac tissues was performed using T1-weighted spin-echo early gadolinium enhancement (EGE) and phase-sensitive inversion recovery late gadolinium enhancement (LGE). A dose of 0.1 mmol/kg of gadobutrol contrast medium was injected for the acquisition of EGE images and another 0.1 mmol/kg for the acquisition of LGE images. Black-blood T2-weighted short tau inversion recovery images, as well as T1/T2 and myocardial extracellular volume fraction (ECV) mapping, were also evaluated. The diagnosis of myocardial inflammation was based on the modified Lake Louise criteria [17].

The brain MRI protocol included spin-echo T1- and T2-weighted imaging, fluid-attenuated inversion recovery (FLAIR) imaging, diffusion-weighted (DW) imaging, susceptibility-weighted (SW) imaging, time-of-flight (TOF) MR angiography, and contrast-enhanced T1-weighted images (1 mm) of areas of abnormality on MRA/TOF. The presence of white matter hyperintensities (WMHs) was determined by visual evaluation, and classified according to their anatomic location.

### 2.3. Statistical Analysis

Statistical analyses were carried out using R version 4.2.0. Normality of continuous variables was evaluated visually using Q-Q plots and/or histograms. Normally distributed variables were compared between patients with IAs and disease controls using independent sample *t*-tests, continuous not-normally distributed variables were compared using Mann–Whitney tests, and categorical/binary variables were compared using chi-square tests or Fisher’s exact tests if appropriate. Multigroup comparisons of age and sex between patients with IAs, healthy controls, and disease controls were performed using Kruskal–Wallis and chi-square tests, respectively. Normally distributed variables are presented as means (standard deviation), continuous not-normally distributed variables are presented as medians (interquartile range), and categorical/binary variables are presented as numbers (%). The relationship between cardiac MRI indices and the presence of WMHs was examined for each variable separately using univariable logistic regression analysis. The association of cardiac MRI parameters with the number of WMHs (lesion burden) was examined using ordinal logistic regression analysis.

## 3. Results

Patients with IAs, disease controls, and healthy controls, respectively, had median (IQR) ages of 45 (39, 51) vs. 53 (40, 57) vs. 43 (37, 44) years (*p* = 0.267); 16 (64%) vs. 7 (23%) vs. 16 (64%), respectively, were women (*p* = 0.001). Patients with IAs were diagnosed with rheumatoid arthritis [14 (56%)], ankylosing spondylitis [5 (20%)], juvenile rheumatoid arthritis [3 (12%)], mixed connective tissue disease including joint involvement [2 (8%)], or enteropathic arthritis [1 (4%)]. Disease controls had various CVDs including coronary artery disease [five (17%)], myocarditis [five (17%)], hypertension [three (10%)], Duchenne muscular dystrophy [three (10%)], and others (Table 1).

No patients showed any focal neurologic deficits or evidence of dementia, memory loss, or cognitive dysfunction. Collectively, no patients had any objectifiable abnormalities in routine cardiovascular clinical evaluation, and standard echocardiography did not demonstrate any systolic dysfunction, regional wall motion abnormalities, or significant valvular disease.

WMHs were detected in ≥1 brain area in 15 (60%) patients with IAs and 17 (55%) disease controls (*p* = 0.907). The majority of patients with IAs had WMHs in subcortical WM [15 (60%)], periventricular WM [15 (60%)], or deep WM [5 (20%)]. Some also had cortical lesions [four (16%)]. Basal nuclear, brainstem, and pontine lesions, as well as mesial temporal sclerosis, were not identified in any patients with IAs. The anatomical localization of lesions was not significantly different between patients with IAs and disease controls (Table 1). Amongst patients with IAs and disease controls with evidence of WMHs, the median (IQR) number of brain lesions was one (1, 2) for both groups (*p* = 0.685). Patients with IAs had significantly higher ECV values compared with disease controls [29 (28, 31) vs. 26 (25, 28), respectively, *p* = 0.004]. In contrast, fewer patients with IAs showed evidence of LGE [9 (36%)] compared with disease controls [21 (67.7%)] (*p* = 0.036), while LGE was significantly more extensive amongst disease controls [4.0% (0.0–6.0%) of LV mass] compared to patients with IAs [0.0% (0.0–4.0%) of LV mass] (*p* = 0.010). Other cardiac MRI findings were largely similar between the two groups (Table 1). Of the healthy controls, two (8%) showed evidence of subcortical WMHs, which was significantly lower compared to patients with IAs (*p* < 0.001).

Amongst patients with IAs, each 0.1 unit increase in the cardiac T2 ratio was associated with an increased probability of WMH occurrence [OR (95% CI): 1.29 (1.05–1.59), *p* = 0.016] (Table 2). A higher cardiac T2 ratio (per 0.1 unit change) and extracellular volume fraction (ECV) were associated with a higher WMH lesion burden in ordinal logistic regression analysis [OR (95% CI): 1.18 (1.05–1.32), *p* = 0.006 and 1.58 (1.04–2.38), *p* = 0.030, respectively] (Table 3). Higher T2 mapping values tended to be associated with a higher brain lesion burden, although this did not reach statistical significance (*p*= 0.057). MRI-derived left/right-ventricular ejection fraction, early/late gadolinium enhancement, and T1 mapping were not associated with the presence of WMHs (Table 2 and Table 3).

## 4. Discussion

Our results illustrate that amongst patients with IAs who reported cardiac symptoms, 60% showed evidence of subclinical WMHs in brain MRI, which was as prevalent in age-matched disease controls with non-autoimmune CVD, but significantly more prevalent compared to age/sex matched healthy controls. Cardiac MRI indices reflecting myocardial oedema and expansion of the cardiac extracellular space were associated with both the presence of brain lesions as well as a greater WMH burden, thus potentially suggesting shared pathophysiologic substrates.

To our knowledge, this is the first study in the literature that investigated the utility of a combined brain–heart MRI examination specifically in patients with IAs. Perhaps the most important finding of our study is the degree to which the presence of IAs, similar to that of non-autoimmune CVD, was associated with a drastically increased presence and burden of WMHs. The age-matched healthy control cohort, in contrast, showed a very low prevalence of WMHs. Most importantly, all study participants were relatively young, with a mean age of 45–50 years. The significance of these findings is two-fold. Firstly, they illustrate that IAs are on equal standing with non-autoimmune CVD regarding an increased risk of brain involvement. Secondly, it can be deduced that both IAs and non-autoimmune CVD may already involve the brain, even at a relatively young age.

We previously reported combined brain and heart MRI findings in a mixed population of patients with autoimmune rheumatic diseases (ARDs) [11], as well as in patients with systemic lupus erythematosus/antiphospholipid syndrome [16]. Collectively, these studies serve to contribute to a growing body of evidence that underscores the potential of autoimmune disease to affect both the heart and the brain.

An important consideration in this context is the clinical significance of the identified brain alterations. Although little is known specifically about patients with IAs, evidence suggests that WMHs in asymptomatic patients with systemic vasculitides may indicate the presence of microangiopathy and may lead to cognitive impairment [18,19]. Cerebral vasculitis has also been reported in patients with SLE [20], Behcet disease [21], and neuro-sarcoidosis [22,23].

According to a systematic literature review, rheumatoid arthritis and systemic lupus erythematosus increase ischemic and hemorrhagic stroke risk by 60–100% relative to the general population [9].

Another important finding of our study is that a higher cardiac T2 ratio and ECV values were associated with a higher WMH lesion burden. This association was identified, to our knowledge, for first time in patients with IAs. As both the T2 ratio and ECV represent indices of myocardial inflammation [17], our findings suggest a close interplay between myocardial inflammation and the development of WMHs. A similar but statistically non-significant trend was identified for T2 mapping, but T1 mapping was not associated with the presence or burden of WMHs. Nevertheless, this may be explained by the considerable number of missing T1/T2 mapping data values for patients with IAs. Lastly, the lack of correlation between ventricular systolic function and WMH was expected, as the ejection fraction represents an index that is influenced relatively late by myocardial inflammation [17]. This suggests that the presence of cardiac inflammation is related to WMH occurrences and supports the need for a combined brain and heart MRI examination, including cardiac tissue characterization, in IAs patients with cardiac symptoms.

In a broader context, the association of elevated systemic inflammation indices with the presence of brain lesions has been reported in a number of investigations in population-based cohorts. Specifically, plasma levels of circulating C-reactive protein (CRP) and interleukin-6 were found to have a genetic correlation with brain SVD [24,25], independently of age and cardiovascular risk factors. Other studies showed that amongst multiethnic, nondemented, old adults, increased circulating inflammatory biomarkers were associated with the presence of infarcts and microbleeds, WMH burden, and progression of WMH [25]. This raises the question of whether an integrated biomarker-based approach could be used to pre-select patients for combined brain–heart MRI examination. Nevertheless, cardiac MRI findings in patients with autoimmune disease tend to be independent of CRP [11,16,26,27]. On the other hand, circulating inflammatory biomarkers were related to cerebrovascular disease only in older people [28]. As such, an approach using CRP-based screening may be challenging. Based on our clinical experience, a more detailed clinical examination, beyond blood inflammatory biomarkers, taking into consideration subtle symptoms that are usually unnoticed by patients and cognitive function tests, should guide patient selection for brain–heart MRI. However, more research is required to refine such a selection process, most likely involving the development of a multi-parameter risk score.

The findings of this investigation have important clinical implications in the risk stratification and management of IAs patients, as these patients are characterized by increased incidence of CVD and early brain damage compared to the normal population without IAs [11]. In parallel to the known additive value of CMR in the evaluation of IAs patients with cardiovascular symptoms [10], this study also demonstrated the additive value of brain MRI, as it identified WMH lesions in the majority of IAs patients. Furthermore, MRI is the only non-invasive imaging modality that can evaluate both the heart and the brain in the same examination without using ionizing radiation. Most importantly, cardiac MRI can perform tissue characterization and is operator-independent and highly reproducible. Considering the adverse effect of WMHs on cognition and the relatively young age of patients with IAs, our findings suggest that combined brain–heart MRI evaluation might be an appropriate diagnostic approach in this patient population.

Lastly, combined brain–heart MRI can play a complementary role in guiding immunomodulatory treatment in IAs patients, as it is known that the timely initiation of immunomodulatory treatment and aspirin, as needed, might attenuate macro- and microvascular CV damage [29,30]. However, the appropriate evaluation of IAs patients with combined heart and brain involvement is still undefined, and no therapeutic guidelines exist for routine treatment evaluation. Large multicenter brain–heart MRI studies with long-term follow up are needed to establish the clinical implications of this approach in the risk stratification and treatment of these patients.

The limitations of this investigation were its small population size, a lack of data regarding circulating inflammatory markers, as well as a lack of detailed neuropsychiatric evaluation using standardized examinations beyond a comprehensive neurologic examination.

## 5. Conclusions

In patients with IAs and cardiac symptoms, the majority showed evidence of subclinical brain lesions that were as prevalent as in age-matched disease controls with non-autoimmune CVD, but more prevalent compared to age/sex matched healthy controls. Myocardial edema was associated with a greater WMH burden, thus potentially suggesting shared pathophysiologic substrates. However, further research is required to determine the clinical impact of these findings.

## Figures and Tables

**Table 1:** Comparison of demographic, clinical, and MRI characteristics between patients with IAs and disease controls with non-autoimmune cardiovascular disease.

Variable	IAs	Disease Controls	*p*-Value
**Group Size**	25	31	N/A
**Demographics**			
Age (Years)	45 [39, 51]	53 [40, 57]	0.266
Female Sex	16 (64.0%)	7 (22.6%)	0.004 *
**Disease Type**			N/A
Coronary Artery Disease		5 (16.1%)	
Myocarditis		5 (16.1%)	
Arrhythmia		3 (9.7%)	
Duchenne Muscular Dystrophy		3 (9.7%)	
Hypertensive Cardiomyopathy		3 (9.7%)	
Atrial Septal Defect		2 (6.5%)	
Non-compaction Cardiomyopathy		2 (6.5%)	
Amyloidosis		1 (3.2%)	
ARVC		1 (3.2%)	
DCM		1 (3.2%)	
MD		1 (3.2%)	
Myocardial Infarction		1 (3.2%)	
Myopericarditis	14 (56.0%)	1 (3.2%)	
PPCM	5 (20.0%)	1 (3.2%)	
Takotsubo Syndrome	3 (12.0%)	1 (3.2%)	
	2 (8.0%)		
Rheumatoid Arthritis	1 (4.0%)		
Ankylosing Spondylitis			
Juvenile Rheumatoid Arthritis			
Mixed Connective Tissue Disease			
Eneteropathic Arthritis			
**Cardiac MRI Findings**			
LVEDV (mL)	129 [101, 147]	127 [108, 163]	0.207
LVESV (mL)	47 [36, 57]	50 [38, 64]	0.364
LVEF (%)	64 [61, 68]	63 [57, 69]	0.458
RVEDV (mL)	115 [90, 136]	133 [111, 161]	0.140
RVESV (mL)	40 [31, 44]	48 [35, 64]	0.048 *
RVEF (%)	65 [61, 70]	61 [55, 64]	0.012 *
EGE	2.7 [2.0, 3.6]	2.5 [2.0, 3.0]	0.557
LGE (% LV Mass)	0.0 [0.0, 4.0]	4.0 [0.0, 6.0]	0.010 *
Native T1 Mapping (ms) **	1056 [992, 1097]	1034 [999, 1057]	0.564
Post-Contrast T1 Mapping (ms) **	397 [351, 405]	420 [370, 437]	0.044 *
T2 Mapping (ms) **	53 [49, 62]	53 [49, 55]	0.515
ECV (%) **	29 [28, 31]	26 [25, 28]	0.004 *
**Normal Value Cut-off Points for Cardiac MRI Variables**			
EGE > 4	5 (20.0%)	5 (16.1%)	0.980
LGE > 0% LV Mass	9 (36.0%)	21 (67.7%)	0.036 *
Native T1 Mapping > 1000 ms **	9 (64.3%)	22 (71.0%)	0.920
ECV > 28% **	9 (64.3%)	7 (22.6%)	0.018 *
T2 Ratio > 1.9 **	13 (52.0%)	19 (61.3%)	0.670
T2 Mapping > 50 ms **	9 (64.3%)	19 (61.3%)	0.999
LVEF < 50%	2 (8.0%)	6 (19.4%)	0.410
RVEF < 55%	1 (4.0%)	2 (6.5%)	0.999
**Brain MRI Findings**			
Any WMH	15 (60.0%)	17 (54.8%)	0.907
Lesion Number	1.0 [0.0, 2.0]	1.0 [0.0, 1.0]	0.598
Subcortical WMH	15 (60.0%)	15 (48.4%)	0.551
Deep WMH	5 (20.0%)	6 (19.4%)	0.999
Periventricular WMH	15 (60.0%)	15 (48.4%)	0.551
Basal Nuclei WMH	0 (0.0%)	1 (3.2%)	0.999
Cortical WMH	4 (16.0%)	1 (3.2%)	0.232
Pontine WMH	0 (0.0%)	0 (0.0%)	0.999
Brainstem WMH	0 (0.0%)	1 (3.2%)	0.999
Mesial Temporal Sclerosis	0 (0.0%)	2 (6.5%)	0.569

IAs, inflammatory arthropathies; ARVC, arrhythmogenic right ventricular cardiomyopathy; DCM, dilated cardiomyopathy; MD, genetic muscular disease; PPCM, peripartum cardiomyopathy; LV/RV, left ventricular/right ventricular; EDV/ESV, end-diastolic volume/end-systolic volume; EF, ejection fraction; EGE/LGE, early/late gadolinium enhancement; ECV, extracellular volume fraction; WMH, white matter hyperintensity. * *p*-value ≤ 0.05, ** variable not available in 11 (44%) patients with IAs.

**Table 2:** Logistic regression for the prediction of the presence of brain lesions amongst patients with inflammatory joint diseases using CMR-derived variables.

Variable	Odds Ratio (95% CI)	*p*-Value
T2 Mapping **	1.13 (0.93–1.38)	0.227
T2 Ratio (per 0.1 unit change)	1.29 (1.05–1.59)	0.016 *
EGE	1.35 (0.80–2.27)	0.258
LGE (% of LV mass)	1.02 (0.76–1.38)	0.880
Native T1 Mapping (per 10 unit change) **	1.00 (0.85–1.18)	0.973
ECV **	1.19 (0.71–2.01)	0.506
LVEF	0.91 (0.78–1.05)	0.196
RVEF	0.99 (0.89–1.11)	0.957

CMR, cardiovascular magnetic resonance; CI, confidence interval; EGE/LGE, early/late gadolinium enhancement; LV, left ventricular; ECV, extracellular volume fraction; LVEF/RVEF, left/right ventricular ejection fraction. * *p* ≤ 0.05, ** variable not available in 11 (44%) patients.

**Table 3:** Ordinal logistic regression for the prediction of the number of brain lesions amongst patients with inflammatory joint diseases using CMR-derived variables.

Variable	Odds Ratio (95% CI)	*p*-Value
T2 Mapping **	1.16 (0.99–1.34)	0.057
T2 Ratio (per 0.1 unit change)	1.18 (1.05–1.32)	0.006 *
EGE	1.05 (0.93–1.17)	0.410
LGE (% of LV mass)	1.05 (0.79–1.41)	0.717
Native T1 Mapping (per 10 unit change) **	1.07 (0.94–1.22)	0.297
ECV **	1.58 (1.04–2.38)	0.030 *
LVEF	0.96 (0.88–1.04)	0.291
RVEF	1.01 (0.91–1.11)	0.919

CMR, cardiovascular magnetic resonance; CI, confidence interval; EGE/LGE, early/late gadolinium enhancement; LV, left ventricular; ECV, extracellular volume fraction; LVEF/RVEF, left/right ventricular ejection fraction. * *p* ≤ 0.05, ** variable not available in 11 (44%) patients.

## Data Availability

The original contributions presented in this study are included in the article. Further inquiries can be directed to the corresponding author(s).
